# Psychological impact of gestational trophoblastic disease: a cross-sectional study

**DOI:** 10.31744/einstein_journal/2025AO1014

**Published:** 2025-04-17

**Authors:** Natália Giovanelli Gaspar, Adrielle Carolina Ferreira Silva, Cristina Laguna Benetti-Pinto, Daniela Angerame Yela

**Affiliations:** 1 Faculdade de Ciências Médicas Universidade Estadual de Campinas Campinas SP Brazil Faculdade de Ciências Médicas, Universidade Estadual de Campinas, Campinas, SP, Brazil.

**Keywords:** Gestational trophoblastic disease, Mental health, Anxiety, Depression, Quality of life, Surveys and questionnaires

## Abstract

**Objective:**

To evaluate the psychological repercussions of gestational trophoblastic disease.

**Methods:**

A cross-sectional study including 100 women (50 with gestational trophoblastic disease and 50 without) was conducted between September 2020 and October 2021. Socio-demographic characteristics, quality of life, depression, and anxiety were evaluated and follow-up was performed at a referral center.

**Results:**

Women with gestational trophoblastic disease had a mean age and body mass index of 28.8±6.4 years and 24.7±5.0kg/m^2^, respectively; the corresponding values for the Control Group were 30.1±6.9 years (p=0.27) and 28.9±4.6kg/m^2^ (p<0.001). The Gestational Trophoblastic Disease Group presented a better general health status (p=0.04) than the Control Group. Among women with gestational trophoblastic disease, 62% and 46% had anxiety and depression, respectively; the corresponding percentages for women without gestational trophoblastic disease were 52% and 24% (p=0.64 and 0.08, respectively).

**Conclusion:**

Multidisciplinary follow-up at a referral center may improve the mental health and quality of life scores of women with gestational trophoblastic disease.

## In Brief

Gestational trophoblastic disease is characterized by the abnormal and excessive proliferation of trophoblast cells, which causes great stress in women owing to pregnancy loss and fear of cancer. Follow-up in referral centers can minimize complications and improve the psychological aspects of women.

## Highlights

■ Gestational trophoblastic disease should be followed up at a referral center.
■ Adequate follow-up improves quality of life for affected women.
■ Multidisciplinary monitoring reduces anxiety and depression in women.

## Graphical abstract

**Figure:**
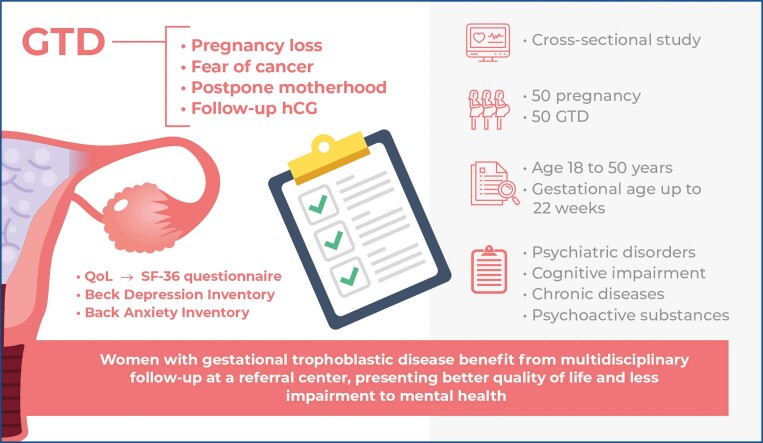

## INTRODUCTION

Gestational trophoblastic disease (GTD) is a heterogeneous group of disorders characterized by the anomalous proliferation of cells that make up the trophoblastic tissue.^1,2^

Medical outcomes related to GTD are well studied and established; however, the effect of this disease on the quality of life (QoL) of patients requires attention. Most studies on the psychological aspects of GTD consider anxiety, depression, impact on sex life, and concern about future fertility in the sense that the disease has a negative impact on them.^3,4^

Despite the low incidence and high cure rates of GTD, the psychosocial consequences of the disease can persist in the long term, even for women with a benign diagnosis, as it is a stressful event characterized by loss of expectancy for a future pregnancy. Even if women did not plan for the pregnancy in which they were diagnosed with GTD, they can be affected by postponement of a future pregnancy until complete remission of the disease, questions on their reproductive future, and the potential risk of the disease evolving into a malignant form. Therefore, the importance of an approach for women with GTD that considers the possible effects on their mental health and QoL should be emphasized.

Pregnancy loss, uterine evacuation surgery, follow-up with continuous monitoring of βhCG levels, probability of chemotherapy, and guidance to postpone future pregnancy tend to be determining factors in the QoL of women with GTD.^5^ However, studies focused on the social, sexual, and other psychological aspects of GTD are scarce.^5-8^

## OBJECTIVE

To evaluate the psychological and social impacts of gestational trophoblastic disease in order to elucidate the effect of this disease on the quality of life of affected women, and thereby contribute to a better approach to follow-up and treatment.

## METHODS

A cross-sectional study including follow-up was conducted at the *Universidade Estadual de Campinas* Gestational Trophoblastic Disease Center from September 2020 to October 2021 with 100 women divided into two groups: 50 women with GTD (GTD Group) and 50 without (Control Group). The women were evaluated using validated questionnaires administered face-to-face, over the phone, and online.

Women aged 18-50 years with an anatomopathological diagnosis of GTD and pregnant women aged 18-50 years with a gestational age of up to 22 weeks comprised the GTD and Control Groups, respectively. Women with psychiatric disorders and cognitive impairment that made it impossible to understand the instruments, those with chronic diseases that could impact their QoL, those who used medications to treat depression or anxiety, and those who used psychoactive substances such as illicit drugs were excluded from both groups.

The variables analyzed were age, race, parity, number of pregnancies and children, education (elementary, high school, and higher), marital status (with or without a partner), professional activity (unemployed or employed), religion, income (calculated with respect to the minimum wage, which was R$ 1,045.00), body mass index (BMI), GTD status (active or in remission), type of GTD (hydatidiform mole (HM) or gestational trophoblastic neoplasia (GTN)), pregnancy planning, time elapsed since diagnosis (months), understanding of GTD, family history of GTD, QoL, depression, and anxiety.

Quality of life was assessed using the SF-36 questionnaire (Short-Form Health Survey), an instrument validated in Brazil and consisting of 36 questions grouped into 8 dimensions: functional capacity, physical capacity, pain, general health status, vitality, social aspects, emotional aspects, and mental health. The final score can vary on a scale of 0 to 100, wherein a higher score indicates a better QoL.^9,10^

Depression was assessed using the Beck Depression Inventory. It consists of 21 items, including symptoms and attitudes, which are scored from 0 to 3. The classifications defined were no (less than 10 points), mild (10-18 points), moderate (19-29 points), and severe (30-63 points) depression.^11^

Anxiety was assessed using the Beck Anxiety Inventory. It comprises 21 items, with scores ranging from 0 to 3, which reflect the symptoms of anxiety. The final score indicates the degree of anxiety and is classified as minimum (0-7 points), mild (8-15 points), moderate (16-25 points), and severe (26-63 points).^12^

All the women included in this study signed an informed consent form before participation. This study was approved by the Research Ethics Committee of *Universidade Estadual de Campinas* (CAAE: 28762819.0.0000.5404; # 3.924.284).

### Statistical analysis

The sample size was calculated to compare the mean Beck depression scores of the two groups (Control and GTD) with estimates obtained from literature; the significance level and power of the study were set at 5% and 80%, respectively.^3,6^ A minimum sample size of 100 women (50 Controls and 50 patients with GTD) was estimated.

The χ^2^ and Fisher’s exact tests were used to analyze the association between categorical variables. The Mann-Whitney U test was used to analyze the association between continuous variables. A probability value (p) of <0.05 was considered statistically significant. SAS version 9.04 (SAS Inc., Cary, NC, USA) was used for all statistical analyses.

## RESULTS

The mean ages of women in the GTD and Control Groups were 28.8±6.4 and 30.1±6.9 years (p=0.27), respectively. The corresponding mean BMI values were 24.7±5.0 and 28.9±4.6kg/m^2^ (p<0.001). In both groups, the majority of women were white and had a partner, a high school education, a professional occupation, and an income above the minimum wage. In both groups, almost 50% of the women had planned their pregnancy (p=0.49). Most women with GTD were primigravidae (p=0.002), whereas most without GTD already had children (p=0.009) (Table 1).

**Table 1:** Clinical and demographic characteristics of women with and without gestational trophoblastic disease

	Control Group (n=50) Mean±SD n (%)	GTD Group (n=50)Mean±SD n (%)	p value
Age (years)	30.1±6.9	28.8±6.4	0.27*
BMI (kg/m^2^)	28.9±4.6	24.7±5.0	<0.001*
Caucasian^#^	28 (57.1)	26 (52.0)	0.60^†^
With partner^#^	34 (69.3)	29 (60.4)	0.01^†^
Education^#^			0.47^†^
Elementary	7 (18.4)	6 (13.3)	
High school	22 (57.8)	23 (51.1)	
Higher	9 (23.6)	16 (35.5)	
Catholic	12 (24.0)	23 (46.0)	0.008^‡^
Primiparous	11 (22.0)	26 (52.0)	0.002^†^
Nulliparous	14 (28.0)	22 (44.9)	0.08^†^
Children (≥1)	28 (56.0)	15 (30.0)	0.009^†^
Employed^#^	28 (70.0)	32 (80.0)	0.30^†^
Income^#^			0.54^‡^
<MW	6 (15.7)	3 (13.6)	
≥MW	34 (84.2)	37 (86.2)	
Pregnancy planning (yes)^#^	17 (41.4)	22 (48.8)	0.49^†^

* Mann-Whitney U test; ^†^ χ^2^ test; ^‡^ Fisher test; ^#^ missing data.

GTD: gestational trophoblastic disease; SD: standard deviation; BMI: Body Mass Index; MW: minimum wage.

The mean gestational age of women in the Control Group was 121.0±4.0 days. Of the women with GTD, 84% had been diagnosed with HM, 60% had been diagnosed with GTD at least six months prior, 64% exhibited remission of the disease, 12% had undergone chemotherapy, and 30% did not understand the disease. No woman had a family history of GTD.

Both groups showed similar QoL results in terms of physical aspects, pain, vitality, social and emotional aspects, and mental health. Women with GTD had better functional capacity (p=0.05) and general health status (p=0.04) than those without GTD (Table 2).

**Table 2:** Evaluation of quality of life, depression, and anxiety of women with and without gestational trophoblastic disease

	Control Group (n=50) Mean±SD n (%)	GTD Group (n=50) Mean±SD n (%)	p value
SF-36 Domains			
Functional capacity	75.0±22.4	82.6±20.5	0.05*
Physical capacity	55.5±45.2	68.1±39.5	0.20*
Pain	69.8±25.2	72.1±24.8	0.63*
General health status	58.6±20.2	65.9±16.0	0.04*
Vitality	60.4±18.8	55.9±20.2	0.16*
Social aspects	75.0±24.7	72.2±22.9	0.44*
Emotional aspects	59.3±41.1	61.3±43.3	0.75*
Mental health	64.1±22.3	60.8±21.1	0.41*
BAI score	10.5±9.8	12.7±11.4	0.32*
0-7	24 (48.0)	19 (38.0)	0.64^†^
8-15	11 (22.0)	16 (32.0)	
16-25	10 (20.0)	9 (18.0)	
26-63	5 (10.0)	6 (12.0)	
BDI score	7.3±7.3	9.8±8.6	0.16*
0-9	38 (76.0)	27 (54.0)	0.08^‡^
10-18	7 (14.0)	16 (32.0)	
19-29	4 (8.0)	6 (12.0)	
30-63	1 (2.0)	1 (2.0)	

* Mann-Whitney U test; ^†^ χ^2^ test; ^‡^ Fisher test.

GTD: gestational trophoblastic disease; SF-36: Short-Form Health Survey; SD: standard deviation; BDI: Beck Depression Inventory; BAI: Beck Anxiety Inventory.

Although there was no significant difference, women with GTD had higher scores for anxiety (GTD Group: 12.7±11.4; Control Group: 10.5±9.8; p=0.32) and depression (GTD Group: 9.8±8.6; Control Group: 7.3±7.3; p=0.16). Among women with GTD, 62% and 46% had anxiety and depression, respectively; the corresponding percentages for those without GTD were 52% and 24% (p=0.64 and 0.08, respectively) (Table 2).

Women with GTD exhibited no significant differences in age, education level, planned pregnancy, and type of GTD, irrespective of whether they had anxiety and depression. Women with anxiety had more children than those without anxiety (p=0.02), but this was not the case with regard to depression (p=0.21) (Table 3).

**Table 3:** Clinical and demographic characteristics of women with gestational trophoblastic disease according to depression and anxiety

	Depression			Anxiety		
	No Mean±SD n (%)	Yes Mean±SD n (%)	p value	No Mean±SD n (%)	Yes Mean±SD n (%)	p value
Age (years)			0.20*			1.00*
<20	0 (0)	1 (4.3)		0 (0)	1 (3.2)	
20-39	27 (100.0)	21 (91.3)		19 (100.0)	29 (93.5)	
≥40	0 (0)	1 (4.3)		0 (0)	1 (3.2)	
Education			0.84*			0.69*
Elementary	4 (16.6)	2 (9.5)		1 (6.2)	5 (17.2)	
High school	12 (50.0)	11 (52.3)		9 (56.2)	14 (48.2)	
Higher	8 (33.3)	8 (38.1)		6 (37.5)	10 (34.4)	
Pregnancy planning			0.10^†^			0.91^†^
Yes	14 (60.8)	8 (36.3)		8 (50.0)	14 (48.2)	
Diagnosis			1.00*			1.00*
Hydatidiform mole	23 (85.1)	19 (82.6)		17 (85.0)	25 (83.3)	
GTN	4 (14.8)	4 (17.4)		3 (15.0)	5 (16.6)	
Children	0.3±0.7	0.6±1.0	0.21^‡^	0.2±0.7	0.6±0.9	0.02^‡^

* Fisher test; ^†^ χ^2^ test; ^‡^ Mann-Whitney U test.

GTN: gestational trophoblastic neoplasia; SD: standard deviation.

## DISCUSSION

In this study, most women with GTD (84%) were diagnosed with HM, which is the benign form of the disease; 60% were diagnosed more than six months prior to the study and 64% exhibited complete remission. These factors may have contributed to a better prognosis and, therefore, to the better scores obtained by the study group in the general health status domains, in which a significant difference was observed, as well as in functional capacity and limitations due to physical aspects, pain, and emotional aspects.

The women in the study had an average age of approximately 30 years; most had an education level of high school and above and an income above the minimum wage. Literature reports that younger women have better functionality and QoL than older women. Furthermore, educational level and economic status are also positively associated with higher QoL of women with GTD.^13^

Although not significantly different from the Control Group, women with GTD had worse scores in the social and mental health domains, suggesting greater interference of issues related to psychosocial aspects in the QoL of these women, in relation to functional aspects.

Literature indicates that more than half of the women diagnosed with GTD have a potential risk of developing psychiatric disorders, especially depression and anxiety. The phase immediately after diagnosis has the greatest emotional impact on women and may persist in the long term. The feeling with the greatest impact seems to be fear, which is primarily related to disease recurrence.^13^

Among the women with GTD in this study, 62% and 46% had anxiety and depression, respectively; another study showed corresponding values of 55% and 18%. Women, in general, have mild anxiety and moderate depression, whereas in this study, most had mild anxiety and depression.^7^

Ireson et al showed that women with GTN exhibit higher rates of depression than those with HM. Considering that the former requires chemotherapy and the latter does not, women with GTN may perceive the disease as more serious and a greater threat to their lives, which is in line with previous studies showing that the need for chemotherapy has a greater impact on mood disorders and increases illness-related stress, as well as feelings of loss, anger, confusion, and disability. An additional cause of great stress in women with GTN is the difficulty in communicating with family and friends about the disease, because it is a little-known condition.^4^ In this study, 16% of the women had GTN, 12% had undergone chemotherapy, and 30% did not understand what GTD was, which must have contributed to the lower scores for women with depression. A previous study also showed that 30% of women had no understanding of GTD and thus exhibited a lower psychological impact.^14^

There was no significant difference in the mean anxiety scores of the control and study groups; this can be attributed to pregnant women having anxiety, mainly related to their own pregnancy.^15^

Literature suggests that the desire and possibility of having children in the future are associated with a good QoL. Studies investigating the impact of children show that childless women tend to experience more intense emotions in the face of early pregnancy loss than those who already have children, likely due to concerns about fertility and future goals.^13^

In this study, the frequency of living children differed significantly among the groups of women; women in the Control Group had more children, which may have contributed to the better scores obtained in the domains of vitality, social aspects, emotional aspects, and mental health, although no significant difference was noted. These results are similar to those of two studies indicating that women with children have a better QoL, which may be related to a lower concern on the reproductive future.^5,7^

However, one study demonstrated that the levels of anxiety and depression among women with GTD were not influenced by the number of children prior to diagnosis; the authors concluded that having children would not be a protective factor for these women to avoid psychological repercussions or improve their QoL.^3^ In contrast, individual comparison of the variables for women with GTD showed greater anxiety in those with more living children.

Growing interest in the wellbeing of women with GTD has promoted a better assessment of the perceived effects of the disease and treatment in the physical, psychological, and social dimensions, thereby improving overall assistance to women. It is recommended that affected women receive medical and psychotherapeutic support. In addition, it is essential to clarify the pathophysiological aspects of the disease and chances of recurrence, and conduct long-term follow-up.^10^

In this study, the mean scores for depression and anxiety among the women in the GTD and Control Groups were similar. This can be explained by the multidisciplinary follow-up to which these women were submitted, in addition to the fact that most of the women evaluated had a benign form of GTD and were in remission.

A limitation of this study is that it does not allow cause-and-effect conclusions to be drawn, as it is a cross-sectional study. This study also did not evaluate women at the beginning of the diagnosis, which could have presented results closer to those in literature. However, it is important to emphasize that, when well monitored, women experience a lower impact of GTD in terms of psychosocial repercussions. Furthermore, this study was conducted during the COVID-19 pandemic period, which may have affected the results.

Hence, it is important to expand research in this area to better understand the psychological problems related to GTD and develop a tool to assess these aspects and direct support, promoting better individual care and psychological adjustment.^13^ Literature on this subject is limited; many studies have sought to compare the psychological and social impacts across age groups, types of diagnosis, or time of disease remission.

Furthermore, the loss of expectation of future pregnancy, fear of recurrence, and the threat of mortality can negatively contribute to sexual function, which compromises the QoL of women with GTD.^16^

## CONCLUSION

Women with gestational trophoblastic disease may have benefitted from multidisciplinary follow-up at a referral center as they presented with quality of life and mental health scores comparable to those of control subjects.
